# Characteristics of soils in selected maize growing sites along altitudinal gradients in East African highlands

**DOI:** 10.1016/j.dib.2015.08.030

**Published:** 2015-09-03

**Authors:** Elijah Njuguna, Mary Gathara, Stanley Nadir, Sizah Mwalusepo, David Williamson, Pierre-Etienne Mathé, Jackson Kimani, Tobias Landmann, Gerald Juma, George Ong’amo, Erastus Gatebe, Bruno Le Ru, Paul-andré Calatayud

**Affiliations:** a African Insect Science for Food and Health (icipe), P.O. Box 30772-00100, Nairobi, Kenya; b Jomo Kenyatta University of Agriculture and Technology (JKUAT), P.O. Box 62000, Nairobi, Kenya; c Kenya Forestry Research Institute (KEFRI), P.O. Box 20412–00200, Nairobi, Kenya; d Department of General Studies, Dar es Salaam Institute of Technology (DIT), P.O. Box 2958, Dar es Salaam, Tanzania; e Institut de Recherche pour le Développement (IRD), UMR-7153 LOCEAN, Université Pierre et Marie Curie, Centre IRD France Nord, 32 Avenue Henri-Varagnat, F-93143 Bondy cedex, France; f Aix-Marseille Université, CNRS, IRD, CEREGE UM34, Aix-en-Provence, France; g University of Nairobi, P.O. Box 30197-00100, Nairobi, Kenya; h UMR Laboratoire Evolution, Génomes, Comportement et Ecologie, groupe IRD, Diversité, Ecologie et Evolution des Insectes Tropicaux, UPR 9034, 22 CNRS, 91198 – Gif-sur-Yvette, France and Université de Paris-Sud, 91405-Orsay, France

**Keywords:** Soil macro and micro elements, Organic carbon content, Soil texture, Soil pH, Cation exchange capacity, Electric conductivity, Water holding capacity, Leaching

## Abstract

Maize is the main staple crop in the East African Mountains. Understanding how the edaphic characteristics change along altitudinal gradients is important for maximizing maize production in East African Highlands, which are the key maize production areas in the region. This study evaluated and compared the levels of some macro and micro-elements (Al, Ca, Fe, K, Mg, Mn, Na and P) and other soil parameters (pH, organic carbon content, soil texture [i.e. % Sand, % Clay and % Silt], cation exchange capacity [CEC], electric conductivity [EC], and water holding capacity [HC]). Soil samples were taken from maize plots along three altitudinal gradients in East African highlands (namely Machakos Hills, Taita Hills and Mount Kilimanjaro) characterized by graded changes in climatic conditions. For all transects, pH, Ca, K and Mg decreased with the increase in altitude. In contrast, % Silt, organic carbon content, Al and water holding capacity (HC) increased with increasing altitude. The research provides information on the status of the physical–chemical characteristics of soils along three altitudinal ranges of East African Highlands and includes data available for further research.

**Specifications Table**

**Table:** 

Subject area	*Agriculture and soil science*

More specific subject area	*Physical and chemical characteristics of soils*
Type of data	*Graph, Table*
How data were acquired	*Inductively coupled plasma atomic emission spectrometry, colorimetric analysis, pH meter, conductivity meter, hydrometer method, “European” maximum water holding capacity method*
Data format	*Analyzed, Interpolated*
Experimental factors	*Soil samples were collected at two depths of 0–25 cm and 25–50 c*m*, and at different altitudes*
Experimental features	*Inverse Distance Weighting* (*IDW*) *interpolation was carried out using a spatial analyst tool within a GIS software (ArcGIS version 10.2) to generate interpolation maps showing the soil properties under study in the three regions. In addition, contours were generated from a 30 m resolution ASTER Digital Elevation Model (DEM)*
Data source location	*CHIESA project (Climate Change Impacts on Ecosystem Services and Food Security in Eastern Africa**)**, icipe, Nairobi, Kenya and KENyan Climate Change and Adaptation research group (KENCCA), IRD, Nairobi, Kenya*
Data accessibility	*Data are available in this article.*

**Value of the data**

**Table:** 

• The data reveal how a combination of temperature and rainfall patterns along an altitudinal gradient modifies edaphic components potentially influencing the amount and quality of maize crops. • The described research is valuable for improvements of soil characteristics by application of the appropriate fertilizers for maize production in the according plots. • The data provide information on the status of the physical-chemical characteristics of soils in maize fields along three altitudinal gradients of East African Highlands and includes data accessible for reuse.

## 1 Data, experimental design, materials and methods

### 1.1 Data

Information was gathered on the edaphic characteristic of maize-cultivated plots along altitudinal gradients of three East Africa Highlands. Studied highlands are the key maize production in the region. Sampled altitudinal ranges encompass several tropical and subtropical regions and thus provide varied patterns of environmental factors such as temperature and rainfall. Coupled with the slope, they provide information on soil leaching processes, soil texture and soil physical–chemical properties. The aim of this research was to evaluate and compare quantities of different soil parameters and elements along three altitudinal ranges in East African highlands, namely Machakos Hills (Ke), Taita Hills (Ke) and Mount Kilimanjaro (Tz).

**Fig. 1:**
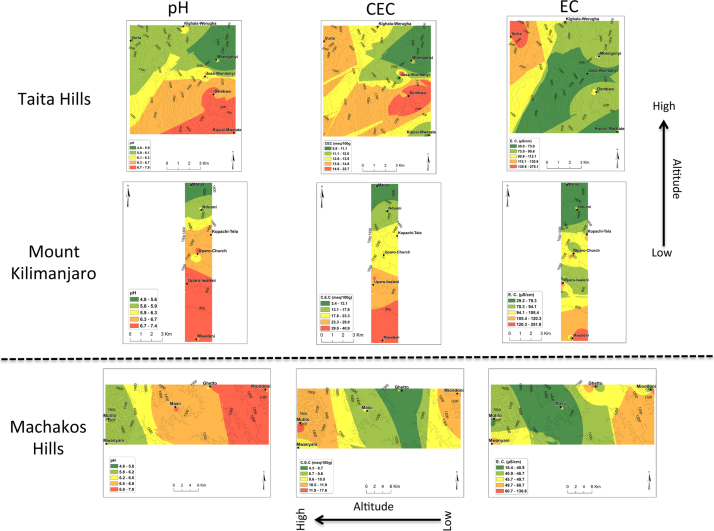
pH, cation exchange capacity (CEC) and electric conductivity (EC) extrapolation maps for Taita hills, mount Kilimanjaro and Machakos hills (for soils sampled at a depth of 0–25 cm).

**Fig. 2:**
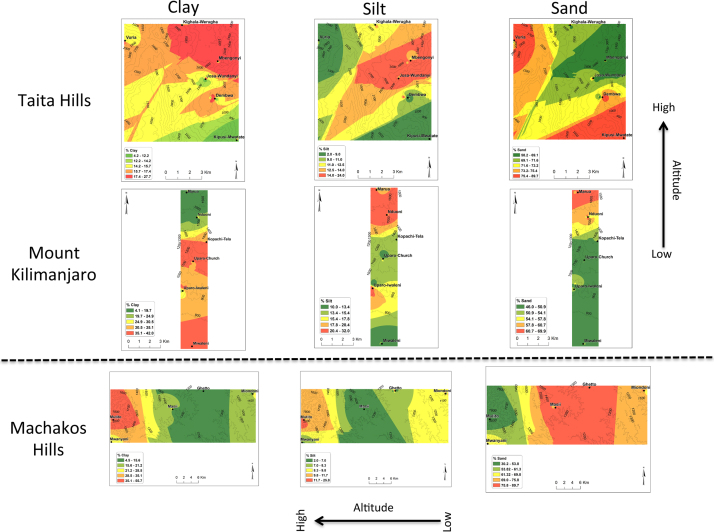
% Clay, % Silt and % Sand extrapolation maps for Taita hills, mount Kilimanjaro and Machakos hills (for soils sampled at a depth of 0–25 cm).

**Fig. 3:**
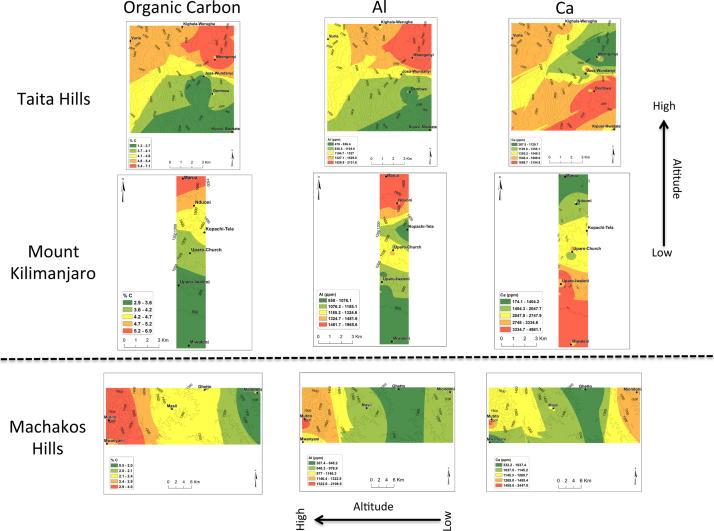
Organic carbon content (C org %), aluminium (Al) and calcium (Ca) extrapolation maps for Taita hills, mount Kilimanjaro and Machakos hills (for soils sampled at a depth of 0–25 cm).

**Fig. 4:**
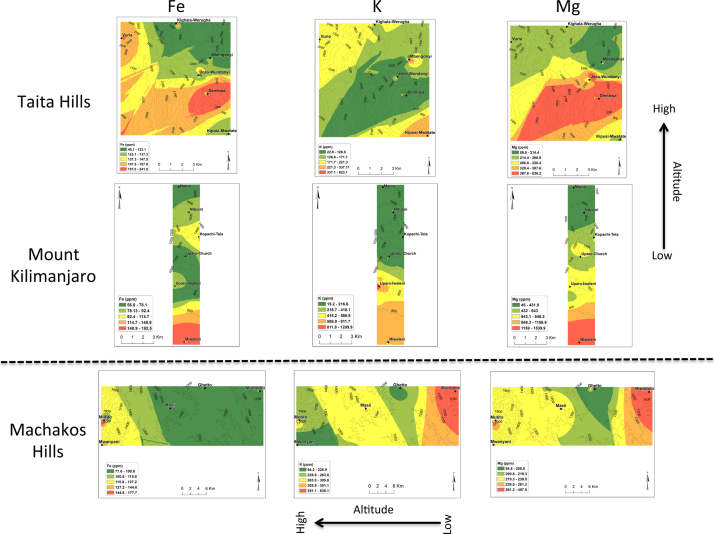
Iron (Fe), potassium (K) and magnesium (Mg) extrapolation maps for Taita hills, mount Kilimanjaro and Machakos hills (for soils sampled at a depth of 0–25 cm).

**Fig. 5:**
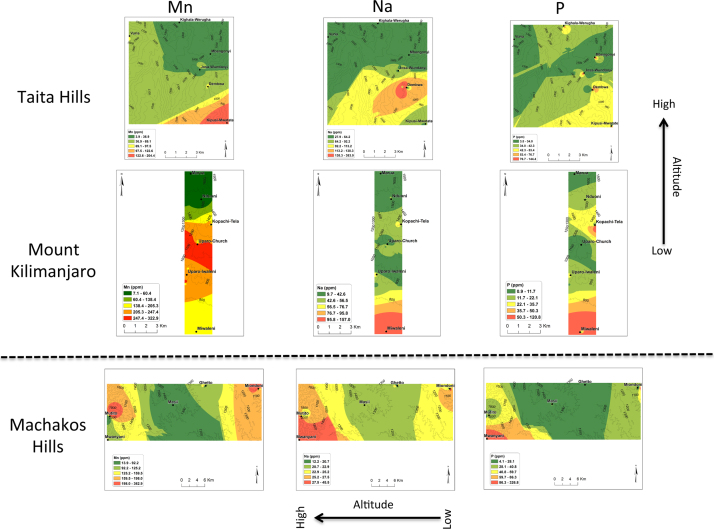
Manganese (Mn), sodium (Na) and phosphorus (P) extrapolation maps for Taita hills, mount Kilimanjaro and Machakos hills (for soils sampled at a depth of 0–25 cm).

**Fig. 6:**
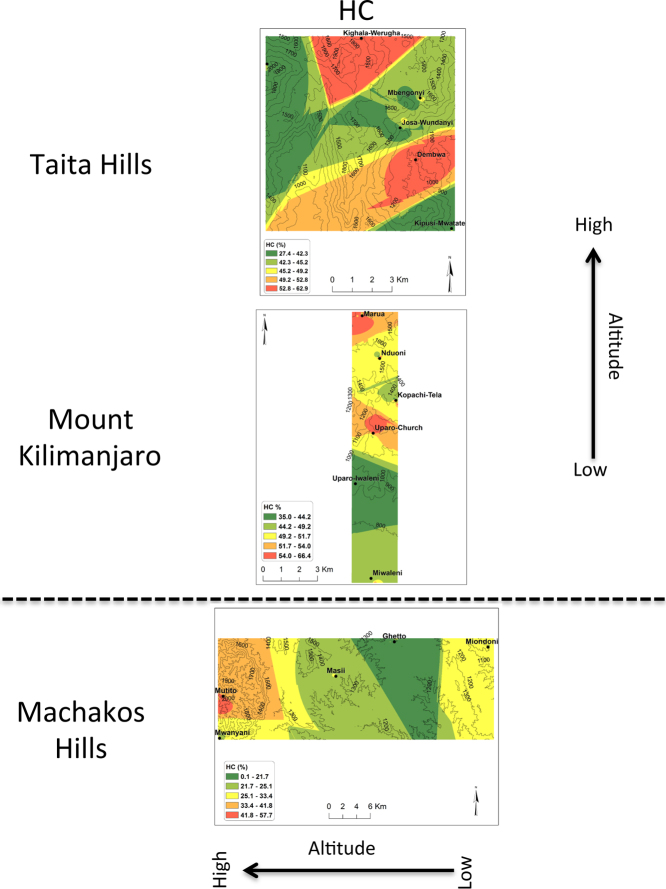
Water holding capacity (HC) extrapolation maps for Taita hills, mount Kilimanjaro and Machakos hills (for soils sampled at a depth of 0–25 cm).

In all the three transects, pH was found to decrease with the increase in altitude (Fig. 1). There was no general trend for cation exchange capacity (CEC) and electric conductivity (EC) as compared to pH. Proportions (%) of silt increased with the increase in altitude in all three transects (Fig. 2). No general trend was observed for the proportions (%) of clay and sand with the increase in altitude. Organic carbon content increased with the increase in altitude in all transects (Fig. 3). Among the soil elements analysed, concentration of Al was found to increase with the increase in altitude whereas Ca, K and Mg decreased with the increase in altitude in the three transects (Fig. 4). For Mn, Na and P no general trends could be observed for all the three transects (Fig. 5). Water holding capacity (HC) increased with the increase in altitude (Fig. 6).

### 1.2 Experimental design, materials and methods

#### 1.2.1 Sites selected

The three altitudinal ranges were established in the Eastern Afromontane Biodiversity Hotspots, along the slopes of (i) the Kilimanjaro volcano and Pangani river basin in Tanzania, (ii) the volcanic Taita Hills in the Taita Taveta County and (iii) the Machakos dome, a strongly weathered paleozoic inselberg of (acidic) metamorphic rocks in Kenya. These locations are characterized by rapid change in altitudinal gradient coupled with changes in temperature and rainfall pattern (Supplementary Table 1).

The Machakos Hills transect consisted of five study sites at different altitudes beginning from the Athi river bridge (1083 m.a.s.l.) and continued along the Machakos–Kitui road to the Iveti Hills (2048 m.a.s.l.). These sites are spatially spread between latitude 01°24.296′ to 01°53.664′ S and longitude 37°17.223′ to 37°63.369′ E (see Table S1). The Taita Hills transect consisted of six study sites between Mwatate (818 m.a.s.l.) and the Vuria hill (1814 m.a.s.l.), spatially spread between latitude 03°39.086′ to 03°47.936′ S and longitude 38°29.575′ to 38°38.001′ E (see Table S1). The Mount Kilimanjaro transect included six study sites varied between Miwaleni (714 m.a.s.l.) and Marua (1683 m.a.s.l.), and spatially spread between latitude 03°30.805’ to 03°42.823′ S and longitude 37°04.501′ to 37°47.245′ E (Supplementary Table 1).

Ten (10) maize plots were sampled in study site with each plot representing one replicate (i.e. *n*=10 for each altitude). GPS coordinates were taken from the middle of each plot. Temperature and relative humidity were recorded hourly using automatic onset ™HOBO data loggers while rainfall was recorded daily using GENERAL^R^ wireless rain gauges placed permanently in one plot per study site of each transect (Supplementary Table 2).

#### 1.2.2 Soil sampling and analysis

Soil samples were taken from each maize plot. Prior to sampling, the upper 1–2 cm layer was removed to avoid any contamination from crop residues. Three to four sub-samples were randomly taken from each plot and pooled. Soil samples were collected at a depth of 0–25 cm and 25–50 cm using a soil auger. Sampling was done in February 2013 in the Machakos Hills transect, and in October 2012 in the Taita Hills and Mount Kilimanjaro transects. The sampling periods coincided with either the off-season or beginning of the planting seasons. The soil sub-samples were bulked and homogenized according to sampling depth to obtain two samples per plot.

Different micro-elements, Al, Ca, Fe, K, Mg, Mn, Na, P (in ppm), organic carbon content (in C org %), pH, CE and CEC in soils were analysed by a private laboratory (Crop Nutrition-Laboratory Services, P.O. Box 66437-00800, Nairobi, Kenya). Mehlich 3 extraction method of Mehlich [1] with diluted ammonium fluoride (NH_4_F) and ammonium nitrate (NH_4_NO_3_) was used for determination of micro-elements. The level of each element was determined by inductively coupled plasma atomic emission spectrometry (ICP-OES). Organic carbon contents were estimated by soil carbon analysis using the method of Walkley and Black [2], a colorimetric analysis after carbon oxidation by acidified dichromate in the presence of sulphuric acid. Since this method does not recover 100% of soil organic carbon, but a lower proportion, a conversion factor of 1.72 was applied. On one hand, the cation exchange capacity (CEC) was calculated using a combination of the pH value and the sum of cations (Ca+Mg+K+Na) while electric conductivity (EC) on the other hand was measured by a 1:2 soil to water extract using a conductivity metre. Soil texture (i.e. % Sand, % Clay and % Silt) of each soil sample was also determined with the hydrometer method of Bouyoucos [3] which estimates the particle size by density. Finally, the “European” maximum water holding capacity (HC) method of Gardner [4] was used to measure the water holding capacity (%) of each soil sample. In this method, each soil sample was saturated with water in a cylinder and the water holding capacity was calculated based on the weight of the water held in the sample *vs*. the sample dry mass (dried at 105 °C for 24 h).
