# Supplementary material for: Characteristics of soils in selected maize growing sites along altitudinal gradients in East African highlands
Source: Data Brief. 2015 Sep 3;5:138–44. doi: 10.1016/j.dib.2015.08.030 (PMC4579291; doi:10.1016/j.dib.2015.08.030)
Supplement: Supplementary file 1 — Supplementary material [file mmc1.doc]

**Table S1** The three transects (Machakos Hills, Taita Hills and Mount Kilimanjaro) with the study sites characterized by altitudinal gradients.

|  | **Localities** | **Latitude** | **Longitude** | **Altitude (m.a.s.l.)** |
| --- | --- | --- | --- | --- |
| **Machakos Hills** | Miondoni | S 01°24.981’ - 01°40.673’ | E 37°37.708’ – 37°63.369’ | 1083 – 1144 |
|  | Ghetto | S 01°24.296’ – 01°40.959’ | E 37°30.563’ – 37°51.670’ | 1134 – 1307 |
|  | Masii | S 01°32.001’ – 01°53.548’ | E 37°17.230’ – 37°28.695’ | 1521 – 1592 |
|  | Mwanyani | S 01°32.115’ - 01°53.664’ | E 37°17.223’ - 37°29.035’ | 1513 – 1792 |
|  | Mutito | S 01°28.438’ - 01°49.575’ | E 37°17.678’ - 37°30.464’ | 1701 – 2048 |
| **Taita Hills** | Kipusi-Mwatate | S 03°47.870’ - 03°47.936’ | E 38°36.726’ – 38°38.001’ | 818 - 871 |
|  | Dembwa | S 03°44.140’ – 03°44.721’ | E 38°36.051’ - 38°36.446’ | 1083 – 1102 |
|  | Josa-Wundanyi | S 03°42.988’ – 03°43.306’ | E 38°35.610’ - 38°35.801’ | 1340 – 1358 |
|  | Mbengonyi | S 03°41.736’ – 03°42.046’ | E 38°36.210’ - 38°36.601’ | 1467 – 1490 |
|  | Kighala-Werugha | S 03°39.086’ – 03°39.265’ | E 38°33.803’- 38°33.896’ | 1678 – 1709 |
|  | Vuria | S 03°40.275’ – 03°40.475’ | E 38°29.575’ - 38°29.656’ | 1797 – 1814 |
| **Mount Kilimanjaro** | Miwaleni | S 03°41.721’ - 03°42.823’ | E 37°45.738’ - 37°45.945’ | 714 - 765 |
|  | Uparo-Iwaleni | S 03°36.675’ - 03°37.460’ | E 37°04.501’ - 37°46.638’ | 891 - 987 |
|  | Uparo-Church | S 03°34.406’ - 03°34.821’ | E 37°46.030’ - 37°45.988’ | 1158 – 1188 |
|  | Kopachi-Tela | S 03°33.008’ - 03°32.916’ | E 37°47.063’ - 37°47.245’ | 1365 – 1402 |
|  | Nduoni | S 03°30.805’ - 03°31.003’ | E 37°46.266’ - 37°46.451’ | 1550 – 1561 |
|  | Marua | S 03°28.615’ - 03°28.821’ | E 37°45.265’ - 37°45.685’ | 1667 – 1683 |

**Table S2** Monthly temperature (°C) and relative humidity (%) (means ± SE), and mean annual rainfall (in mm) along the transects (Machakos Hills, Taita Hills and Mount Kilimanjaro).

|  | **Localities** | **Altitude (m.a.s.l.)** | **Average daily temperature** | **Average nightly temperature** | **Relative humidity over the day** | **Relative humidity over the night** | **Annual rainfall** |
| --- | --- | --- | --- | --- | --- | --- | --- |
| **Machakos Hills^1^** | Miondoni | 1083 – 1144 | n.d. | n.d. | n.d. | n.d. | 744 |
|  | Ghetto | 1134 – 1307 | 27.2 ± 0.7 | 18.7 ± 0.3 | 57.2 ± 3.1 | 87.4 ± 4.0 | 854 |
|  | Masii | 1521 – 1592 | 25.2 ± 0.1 | 19.2 ± 0.4 | 67.9 ± 0.9 | 95.2 ± 1.2 | 845 |
|  | Mwanyani | 1513 – 1792 | 26.1 ± 0.8 | 18.3 ± 0.3 | 56.0 ± 3.7 | 84.5 ± 3.0 | 788 |
|  | Mutito | 1701 – 2048 | 22.4 ± 0.9 | 15.3 ± 0.4 | 65.7 ± 3.4 | 93.5 ± 2.1 | 1192 |
| **Taita Hills^2^** | Kipusi-Mwatate | 818 - 871 | 27.3 ± 0.5 | 20.5 ± 1.4 | 56.7 ± 2.2 | 83.7 ± 1.7 | 1010.4 |
|  | Dembwa | 1083 – 1102 | 25.6 ± 0.7 | 18.5 ± 0.3 | 62.8 ± 1.7 | 92.7 ± 1.5 | 1387.2 |
|  | Josa-Wundanyi | 1340 – 1358 | 21.4 ± 0.6 | 18.1 ± 0.5 | 71.5 ± 2.5 | 91.7 ± 0.9 | 1526.4 |
|  | Mbengonyi | 1467 – 1490 | 21.3 ± 0.5 | 16.6 ± 0.4 | 74.5 ± 2.4 | 90.4 ± 2.1 | 1544.4 |
|  | Kighala-Werugha | 1678 – 1709 | 20.1 ± 0.5 | 15.8 ± 0.4 | 81.0 ± 2.1 | 95.1 ± 1.8 | 1648.8 |
|  | Vuria | 1797 – 1814 | 19.4 ± 0.7 | 15.1 ± 0.4 | 76.4 ± 2.0 | 94.6 ± 1.7 | 1706.4 |
| **Mount Kilimanjaro^2^** | Miwaleni | 714 - 765 | 28.3 ± 0.8 | 22.1 ± 0.4 | 52.5 ± 2.6 | 74.9 ± 1.8 | 865.2 |
|  | Uparo-Iwaleni | 891 - 987 | 25.2 ± 1.2 | 20.3 ± 1.1 | 62.2 ± 2.0 | 85.5 ± 3.0 | 1054.8 |
|  | Uparo-Church | 1158 – 1188 | 23.3 ± 0.4 | 18.3 ± 0.2 | 66.8 ± 2.3 | 80.9 ± 3.2 | 1641.6 |
|  | Kopachi-Tela | 1365 – 1402 | 21.8 ± 0.8 | 17.7 ± 0.4 | 72.9 ± 3.4 | 90.7 ± 1.1 | 2158.8 |
|  | Nduoni | 1550 – 1561 | 21.0 ± 0.7 | 16.3 ± 0.4 | 74.3 ± 3.1 | 92.4 ± 1.9 | 2198.4 |
|  | Marua | 1667 – 1683 | 20.0 ± 0.6 | 15.0 ± 0.3 | 78.4 ± 2.0 | 96.5 ± 0.7 | 1852.8 |

n.d.: not determined; for the Machakos Hills the average annual rainfall was derived from WorldClim (interpolations of observed data, representative of 1950-2000) data set. Further information can be found at http://www.worldclim.org/

^1^recorded from January to March 2013;

**^2^**recorded from June 2012 to June 2013.
